# Mechanical Properties of Bio-Printed Mortars with Bio-Additives for Green and Sustainable Construction

**DOI:** 10.3390/ma18143375

**Published:** 2025-07-18

**Authors:** Sotirios Pemas, Dimitrios Baliakas, Eleftheria Maria Pechlivani, Maria Stefanidou

**Affiliations:** 1 Centre for Research and Technology Hellas, Information Technologies Institute, 6th km Charilaou-Thermi Road, 57001 Thessaloniki, Greece; sopemas@iti.gr; 2 Laboratory of Building Materials, School of Civil Engineering, Aristotle University of Thessaloniki, 54124 Thessaloniki, Greece; dbaliakas@gmail.com

**Keywords:** 3D printing, additive manufacturing (AM), 3D-printed mortars, sustainability, green manufacturing, bio-products, building materials, mechanical properties, sustainable construction

## Abstract

Additive manufacturing (AM) has brought significant breakthroughs to the construction sector, such as the ability to fabricate complex geometries, enhance efficiency, and reduce both material usage and construction waste. However, several challenges must still be addressed to fully transition from conventional construction practices to innovative and sustainable green alternatives. This study investigates the use of non-cementitious traditional mixtures for green construction applications through 3D printing using Liquid Deposition Modeling (LDM) technology. To explore the development of mixtures with enhanced physical and mechanical properties, natural pine and cypress wood shavings were added in varying proportions (1%, 3%, and 5%) as sustainable additives. The aim of this study is twofold: first, to demonstrate the printability of these eco-friendly mortars that can be used for conservation purposes and overcome the challenges of incorporating bio-products in 3D printing; and second, to develop sustainable composites that align with the objectives of the European Green Deal, offering low-emission construction solutions. The proposed mortars use hydrated lime and natural pozzolan as binders, river sand as an aggregate, and a polycarboxylate superplasticizer. While most studies with bio-products focus on traditional methods, this research provides proof of concept for their use in 3D printing. The study results indicate that, at low percentages, both additives had minimal effect on the physical and mechanical properties of the tested mortars, whereas higher percentages led to progressively more significant deterioration. Additionally, compared to molded specimens, the 3D-printed mortars exhibited slightly reduced mechanical strength and increased porosity, attributable to insufficient compaction during the printing process.

## 1. Introduction

Construction using 3D printing technology has opened a new field in the industry, offering various advantages such as the fabrication of complex geometries, enhanced construction precision, cost efficiency, reduced construction time, minimized labor accidents, and the use of automation technologies for improved accuracy [1,2,3]. Inseparable from these advantages are the limitless possibilities for utilizing innovative materials with various mixture combinations, exploring new materials to improve durability and mechanical performance, and investigating eco-friendly options for green building applications [4,5]. Furthermore, with the automation enabled by 3D printing, constructing both simple and complex structures becomes easier compared to traditional cast-in-place methods, requiring less material, which leads to reduced CO_2_ emissions and minimized construction waste [1,6,7]. Three-dimensional printing is an advanced manufacturing technology with broad applications across multiple sectors, enabling the production of new products, structural components, and even entire building constructions using specially developed materials. These materials often incorporate various additives, including by-products from industries such as agriculture and automotive. Examples include powdered wood or non-recyclable tire residues [8,9]. Through additive manufacturing, these waste materials can be transformed into valuable additives that enhance the mechanical and functional properties of composite materials, supporting both material innovation and sustainability. Regarding the use of resources, the sustainability of 3D printing stems from reduced waste during construction, as well as the decreased need for transportation since structures often consist of multiple parts that are typically transferred in smaller sections and assembled on-site. Three-dimensional printing has demonstrated the potential to reduce construction waste by up to 60%, cut production time by 70%, and lower labor costs by 80% [10,11].

In addition to the advancements offered by 3D printing technologies for achieving green manufacturing, the traditional construction sector can further enhance sustainability by incorporating bio-based building materials. Until today, numerous studies have referenced the integration of artificial fibers in concrete mortars [10,12]. However, these fibers are not eco-friendly and require significant energy to produce [10]. For green and sustainable construction, natural fibers offer a viable alternative, enhancing structural, thermal, and energy performance [13,14]. Fibers such as flax, hemp, jute, sisal, kenaf, and bamboo show promising results due to their unique properties and availability [15,16]. The literature highlights significant improvements in tensile, flexural, impact, and thermal performance when using natural fibers in 3D printing mortars for construction [10,17]. Promising results highlight the potential of biodegradable natural materials, such as corn cob, a by-product of the corn industry. Corn cob has shown great potential as a sustainable and versatile building material. It is commonly used for producing geopolymer concrete, an application that has gained traction as a cheaper and more viable alternative to conventional Portland cement concrete. Furthermore, corn cob finds use in clay brick production and as reinforcement in composite materials. Its versatility underscores its potential in eco-friendly construction applications [18,19].

The integration of natural products is gaining more ground in the new era of construction, especially as the European Green Deal aims for the EU to be climate-neutral by 2050 [20]. Reducing CO_2_ emissions in the construction sector is crucial, as it is a major source of emissions, both direct (e.g., from cement production, generators, and construction machinery) and indirect (e.g., from transportation requirements) [21,22]. Benmansour et al. [23] demonstrated the use of date palm fibers as an insulating material to reduce heat loss in buildings. Experimental results showed improved insulation and reduced thermal conductivity, though higher fiber content decreased mechanical strength. The optimal fiber percentage, where mechanical strength remains at desirable levels, can be suitable for energy-efficient buildings. Akemah et al. [24] explored the development of natural fiber-based mixtures for 3D printing by using biopolymer binding agents, such as locust bean gum, alginate, and cellulose, along with natural fibers of wheat straw and hemp. These mixtures were designed to enhance carbon storage and thermal resistivity while also demonstrating their suitability for 3D printing applications. Juárez et al. [25] studied the reinforcement of Portland cement mortars with Agave lechuguilla fibers and demonstrated their high tensile strength. Moreover, the proposed paraffin protective treatment effectively reduced fiber water absorption while preserving tensile strength even after a year of exposure to humid and alkaline conditions. Bong et al. [26] investigated the use of wollastonite microfibers as reinforcement in 3D concrete printing (3DCP) to enhance the flexural strength of 3D-printed geopolymer concrete. The addition of 10% wollastonite increased flexural strength by 54% while maintaining comparable compressive strength. The printed geopolymer also exhibited increased flexural strength in the Y- and Z-directions, whereas the strength in the X-direction (parallel to the layer interface) remained unchanged. Additionally, thixotropy was improved, enhancing shape retention and buildability. Ali et al. [27] evaluated the effect of coconut fibers as reinforcement to enhance concrete properties. Results showed a significant improvement in flexural toughness, with the best mechanical and dynamic performance observed at a 5 cm fiber length and 5% content. Additionally, the investigation of the damping ratio and fundamental frequency showed that higher fiber content led to increased damping, emphasizing the need for further research into the use of these fibers in anti-seismic applications. Most of the aforementioned references on bio-based mixtures highlight their ongoing development to identify the most suitable formulations for future building applications, particularly in terms of flexural and compressive strength to define their mechanical durability. Moreover, an important technique for assessing the durability performance of these bio-modified mortars is freeze–thaw testing under cyclic environmental conditions, which provides valuable insights into their performance across varying climates [28,29,30].

While numerous studies have explored the use of natural fibers in traditional cast-in-place construction, there is a significant gap in research on their integration into 3D concrete printing and 3D printing with building materials in general [16]. This study introduces a methodology to demonstrate the potential of combining green manufacturing and 3D printing technologies to advance sustainable and environmentally friendly construction practices. The objective is to revisit traditional building materials, which have been largely replaced, and reassess their potential as eco-friendly alternatives. This study investigates the impact of incorporating two bio-products, black pine (Pinus Nigra) and Mediterranean cypress (Cupressus sempervirens) shavings, to the mechanical and physical properties of traditional lime–pozzolan mortars. The selection of these bio-products and mortars is based on their regional availability, their usage throughout history in construction [31], and the relative lack of research on them compared to that on other mortars like cement and other bio-products such as straw and hemp. Additionally, all mixtures developed were specifically designed for use in 3D printing construction, particularly with Liquid Deposition Modeling (LDM) technology.

The paper is structured as follows: Section 2 outlines the materials and methods used in this study, including the selected raw materials for the developed mortars, the bio-products used as additives, the 3D printing technique applied, and the equipment used for material and mortar characterization. Section 3 presents the experimental results on the physical and mechanical properties of the mortars. Finally, Section 4 concludes the study and highlights the key findings of the conducted research.

## 2. Materials and Methods

### 2.1. Selected Materials

**Figure 1:**
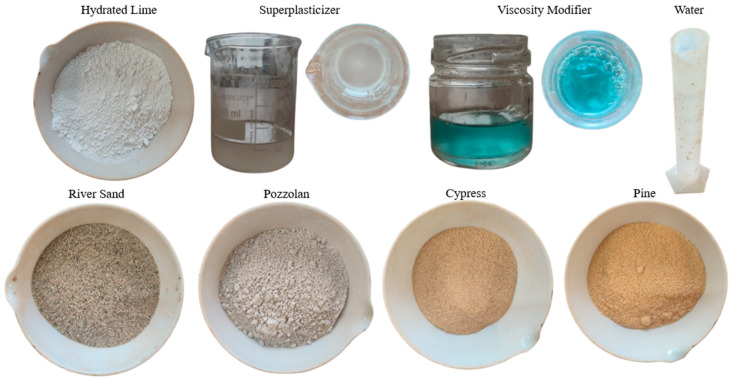
The materials of the present study.

The mortar mixtures in this study were prepared using hydrated lime and natural pozzolan as the primary binders, combined with fine river sand as the aggregate. The lime was commercially available CL90 and the natural pozzolan was also available in the market, while the river sand of silicic origin was sieved in order to remove grains with a size above 1 mm, to avoid clogging during the 3D printing process. The properties of the materials used are presented in Table 1. To further ensure the printability of the mixtures, a polycarboxylate-based superplasticizer (Master Glenium 11, BASF, Ludwigshafen, Germany) and a viscosity modifier (Stabilizer 4R, SIKA, Baar, Switzerland) were incorporated. Finally, black pine and cypress wood shavings, locally sourced from carpentry workshops, were utilized as additives at 1%, 3%, and 5% of the binder weight. Before inserting them in the mixtures, the shavings were sieved as well to remove pieces larger than 0.5 mm to avoid clogging. Figure 1 illustrates the materials used in the present study.

Before developing the mortars, testing was conducted for the binders and the wood shavings. The density of the wood shavings and the binders was determined via the gas pycnometer Ultrapyc 3000 (Anton Paar, Graz, Austria). The composition of the binders was measured in the lab for a previous study [32] with X-ray fluorescence (XRF) using a Bruker TIGER S8 (Karlsruhe, Germany) and their particle size was measured via Laser Diffraction (LD) with a Malvern Panalytical Mastersizer 2000 (Malvern, UK).

### 2.2. Development of Mortars

The mortars were prepared using an automatic programmable mixer, the CONTROLS 65-L0006/AM (Liscate, Milan, Italy), featuring a planetary rotation system for its working body. The specimens were produced according to EN1015-11 [33] with dimensions of 40 mm × 40 mm × 160 mm. First, the superplasticizer, the viscosity modifier, and pre-weighted water were added to the mixer bowl. Then, the binders (hydrated lime and pozzolan) and additives (pine and cypress shavings) were mixed at low speed for 30 s, followed by the gradual addition of river sand during mixing. Subsequently, the mixer was set to high speed for 60 s.

The workability of the mortars was measured as per EN 1015-3 [34], using the flow table test. To ensure extrudability, the mortar was placed in a conical mold to maintain its shape. After removing the mold, the table was dropped 15 times, resulting in an expanded mortar diameter of 17 ± 1 cm. It was observed in the workability tests that mixtures with an expanded diameter larger than 18 cm were too fluid to maintain the desired shape during 3D printing. Conversely, mixtures with an expanded diameter smaller than 16 cm were too rigid and could not flow properly from the 3D printer’s extruder, leading to nozzle clogging. The water content of each mixture was slightly adjusted in order to meet this workability standard.

Table 2 presents the composition details of each mortar developed and examined in this study.

### 2.3. Design of the Specimens

**Figure 2:**
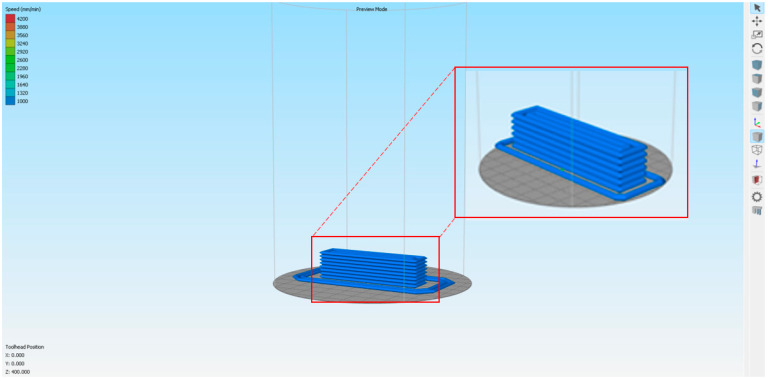
The 3D model used in the printing process.

All specimens were designed using SOLIDWORKS^®^ CAD Software (2022 SP2.0 Professional version) and processed to generate G-code using Simplify3D (Version 5.1.2). The specimens had dimensions of 40 mm × 40 mm × 160 mm, which matched those of the molded samples, aligning with standard testing protocols and facilitating meaningful comparisons with molded ones. Additionally, the selected dimensions supported the printing strategy, as the entire specimen was printed in one go with a continuous line, avoiding infill patterns that could potentially deteriorate the final specimen’s quality. Figure 2 illustrates the printing strategy set in the Simplify3D slicer.

### 2.4. Three-Dimensional Printing of the Developed Mortars

The mortar mixtures were prepared according to EN 1015-11. Liquid Deposition Modeling (LDM) technology was employed in the present study for specimen fabrication. For this technology, the feedstock materials must have a paste-like consistency, as described in the previous section, with specific workability and an expanded diameter of 17 cm, as well as consistent viscosity. The fresh mortar was manually inserted into the printer’s feedstock container, which was then shaken on a vibration table for 45 s to remove any encapsulated air and voids. The container was sealed with a cap, and with the help of compressed air, the material was directed into the extruder. The pressure applied to the cap was set to 0.15 MPa, supplied by an air compressor connected to the top of the container. The extruder, which featured a screw mechanism, pushed the mortar through the nozzle during the 3D printing process. The nozzle used in this study had a 10 mm diameter, and the layer height was set at 5 mm. All specimens were printed at room temperature.

A total of 42 specimens were printed using the aforementioned printing procedure. Specifically, 6 specimens were printed for each different mortar.

**Figure 3:**
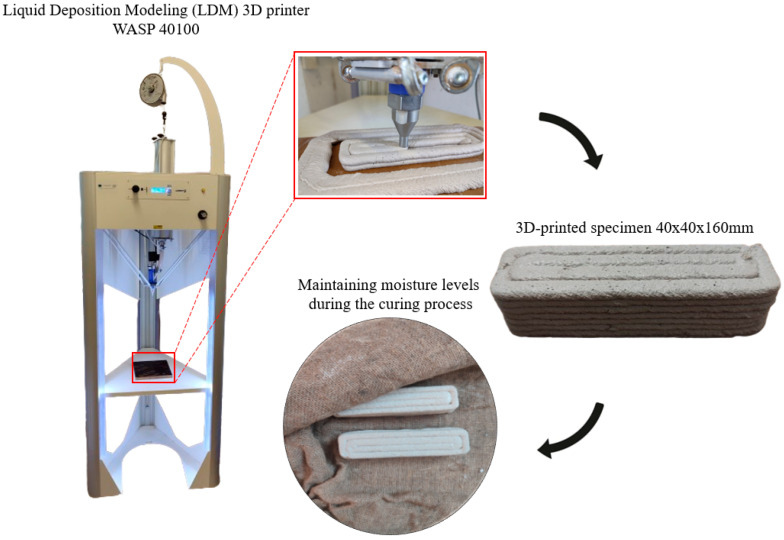
Three-dimensional printing process and specimen moisture maintenance method.

Figure 3 presents the WASP LDM 3D printer along with a schematic representation of the printing process for each specimen. Initially, the specimens were 3D-printed, and after solidifying for one day, they were wrapped in wet burlap and stored at room temperature (24 ± 2 °C) with 95% relative humidity for 28 days. Afterwards, the specimens were removed from the burlaps and stored in laboratory conditions. This curing method was implemented to minimize the risk of cracking due to shrinkage during the drying phase of the mortars.

### 2.5. Molded Specimens—Casting Procedure

**Figure 4:**
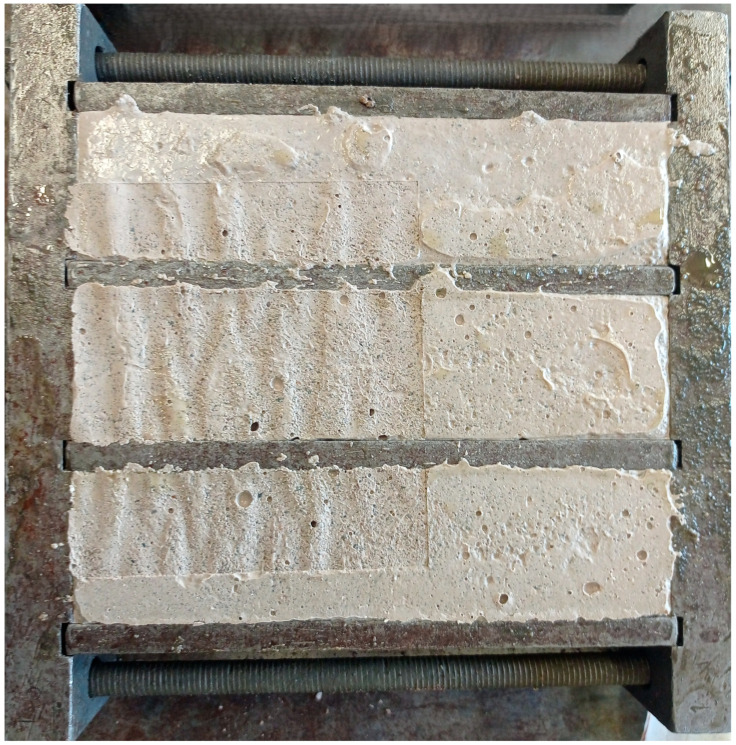
The cast specimens within the metal molds.

Lubricated prismatic metal molds (40 mm × 40 mm × 160 mm) were filled with the fresh mortar mixture to create the molded specimens. To eliminate air bubbles and voids, the filled molds were shaken on a vibration table for 45 s. After two days, the specimens were removed from the molds and cured under the same conditions as those of the 3D-printed ones, wrapped in wet burlap, and stored at room temperature (24 ± 2 °C) with 95% relative humidity. Figure 4 demonstrates the molding of specimens into their molds.

### 2.6. Conducted Measurements

#### 2.6.1. Physical Properties

The porosity, water absorption, and specific gravity of the specimens were measured in accordance with RILEM CPC 11.3 [35]. Following this standard, oven-dried halves of specimens obtained after flexural testing were submerged in water under vacuum conditions for 24 h. However, due to significant cracks forming within the samples under vacuum after 28 days of curing, results were only obtained after 90 days of curing.

The density of the specimens was measured utilizing a gas pycnometer, Ultrapyc 3000 (Anton Paar, Graz, Austria).

#### 2.6.2. Flexural and Compressive Strength

**Figure 5:**
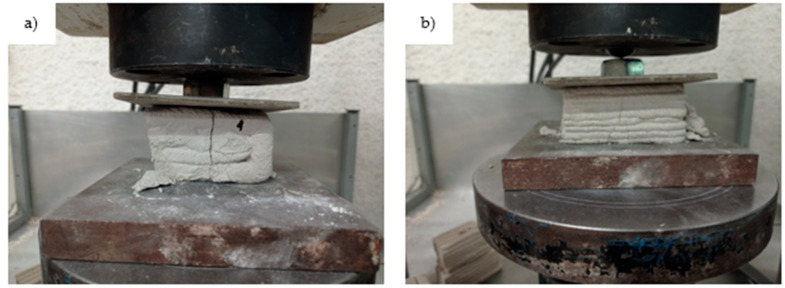
Testing the compressive strength of 3D-printed specimens (**a**) parallel and (**b**) perpendicularly to the printed layers.

The flexural and compressive strength of the specimens were tested using a computer-controlled WAW-300E Universal Testing Machine (UTM), utilizing the MaxTest software (Version 5.7, Physical Test Solutions), in accordance with EN 1015-11:2019 [33]. Compressive strength testing was performed on specimen halves remaining after the flexural testing. To account for the inherent anisotropy of 3D-printed materials, compressive loads were applied in two directions: parallel and perpendicularly to the printed layers (Figure 5). This methodology not only evaluated the printed prism’s compressive strength but also assessed interlayer cohesion, an important factor in 3D-printed materials. Flexural strength was only measured perpendicular to the layers, as testing parallel to the layers caused the gradual delamination of the printed specimens.

#### 2.6.3. Stereoscopic Analysis

After flexural testing, one half of each specimen, both 3D-printed and molded, was examined under a Leica (Wetzlar, Germany) M10 stereo microscope and the images obtained were further analyzed via the Phases and Pores plugin of the DHS Image Database software (Version 2.5FEM, Dietermann and Heuser Solution GmbH, Greifenstein, Germany). This analysis aimed to assess bio-product, aggregate, and pore dispersion within the mixture, while locating microcracks and other structural defects.

## 3. Results

### 3.1. Physical Properties

**Figure 6:**
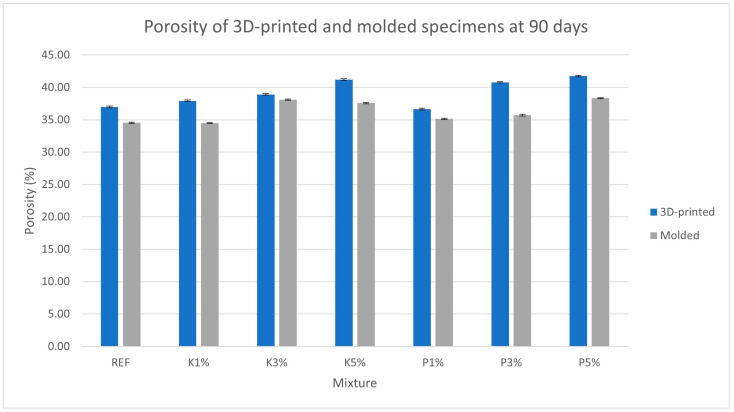
Porosity (%) of the 3D-printed and molded specimens.

**Figure 7:**
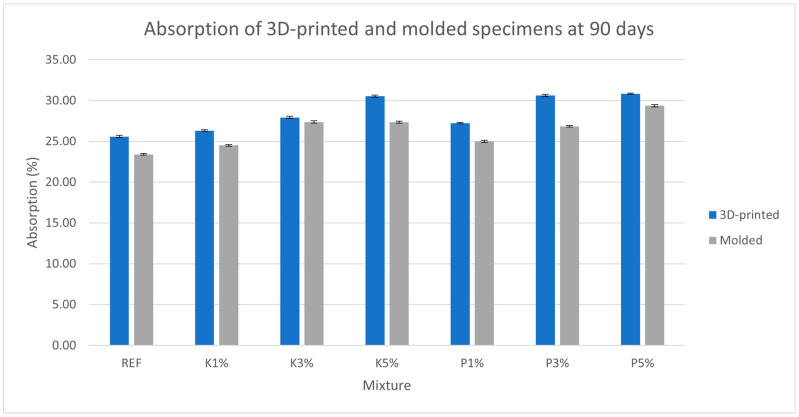
Absorption (%) of the 3D-printed and molded specimens.

**Figure 8:**
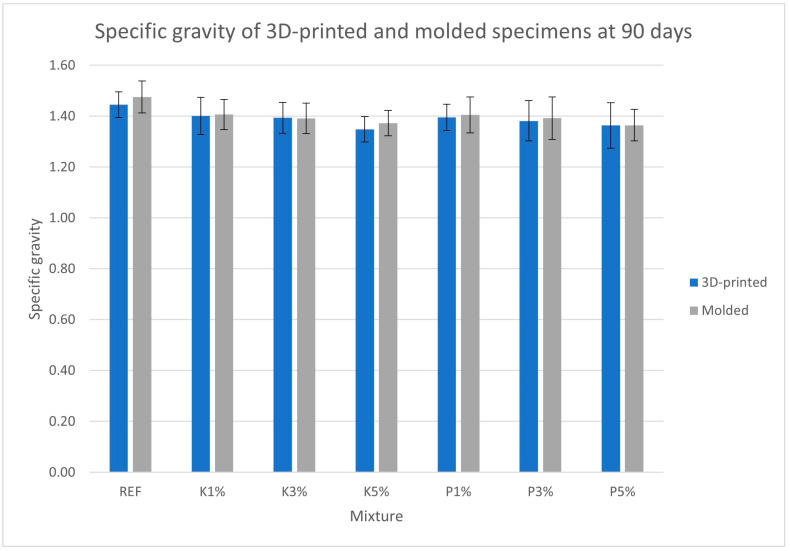
Specific weight of the 3D-printed and molded specimens.

**Figure 9:**
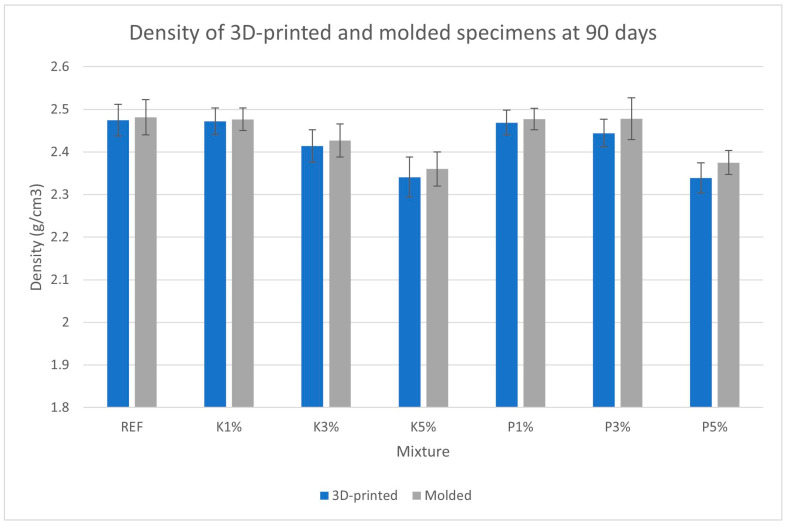
Density of the 3D-printed and molded specimens.

Figure 6, Figure 7, Figure 8 and Figure 9 present the measured porosity percentage, absorption percentage, specific weight, and density of the 3D-printed and molded specimens at 90 days.

As shown in the results, both open porosity and water absorption increased in molded and 3D-printed specimens with the incorporation of wood shavings. This increase may be attributed to the required adjustments made to the mixture’s water content in order to maintain printability. Additionally, 3D-printed specimens exhibited marginally higher porosity and absorption (~8% increase on average for both) compared to their molded counterparts, despite using identical mixtures and preparation methods. This suggests that the printing process by itself, due to the layer-by-layer deposition method, trapped air between filaments and introduced additional voids into the structure.

The specific weight and density results follow an inverse trend with that of porosity and absorption, progressively decreasing as bio-product percentage increases. This trend may be related to the lower density of the organic additives compared to the lime pozzolan binder and the aggregates, as well as the aforementioned increase in porosity. Again, 3D-printed specimens exhibited slightly lower densities (~1% reduction) than their molded counterparts, consistent with the observed porosity differences.

### 3.2. Flexural Strength

**Figure 10:**
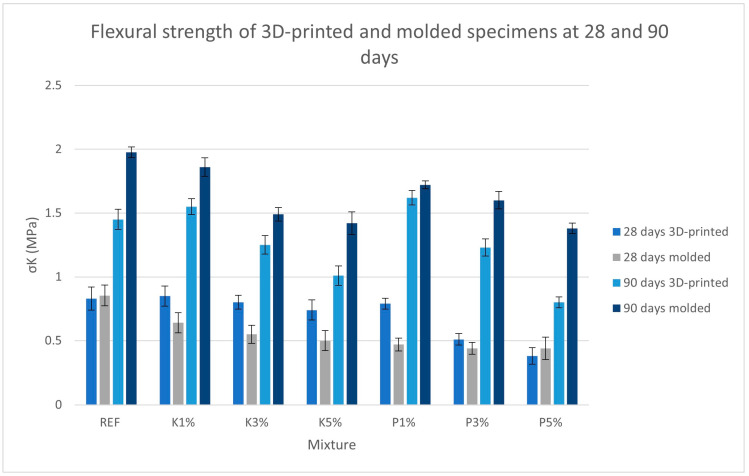
Flexural strength of 3D-printed and molded specimens.

Figure 10 presents the test results for flexural strength at 28 and 90 days.

The wood additives moderately affected flexural strength, with increasing degradation for higher concentrations of additives. Cypress mixtures showed steady, progressive strength loss with increasing content, while mixtures with the inclusion of pine wood shavings showed a more pronounced drop-off when the additive was introduced at concentrations higher than 1%. At the 90-day mark, both additives greatly reduced strength at 5% additive inclusion, with pine causing more severe degradation in printed specimens (~60% reduction from P1% to P5%) than in cypress (~35% reduction from K1% to K5%).

The most notable observation from the graph is the clear divergence between 3D-printed and molded specimens at 28 and 90 days. At 28 days, 3D-printed specimens mostly outperformed their molded counterparts by an average of 13%, perhaps due to the higher porosity and inherently rougher surfaces leading to a higher exposed surface area and, therefore, faster hydration. However, it seems this advantage was lost at the 90-day mark, as molded specimens moderately surpassed 3D-printed ones by an average of 29% (in all but the P1% mixture, where the 3D-printed strength of 1.62 MPa nearly matched the molded strength of 1.72 MPa). This reversal implies that while 3D printing may have accelerated early hydration to an extent (an important factor in traditional mortars), in the long term, the molded specimens developed overall higher strength because of their lower porosity and absence of discontinuities such as layers.

### 3.3. Compressive Strength

**Figure 11:**
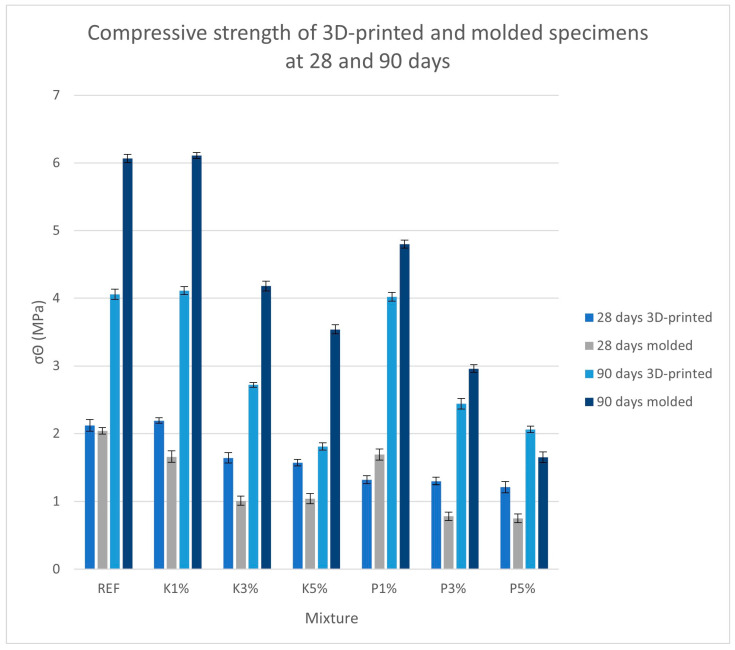
Compressive strength of 3D-printed and molded specimens.

**Figure 12:**
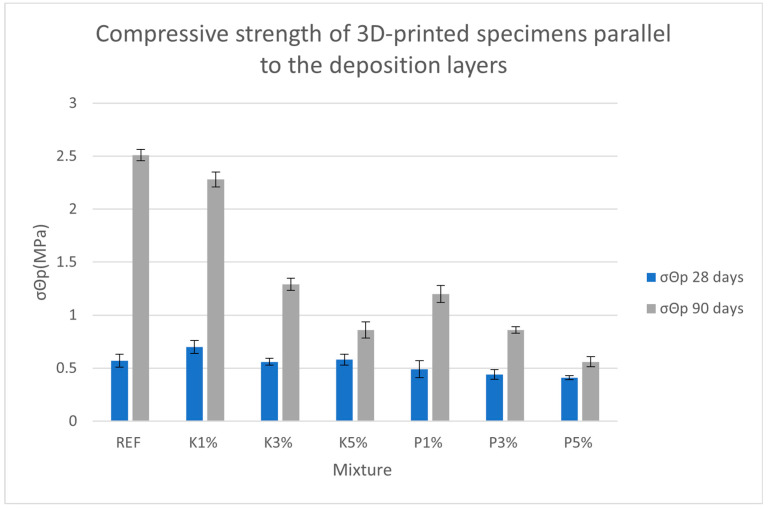
Compressive strength of 3D-printed specimens parallel to the deposition layers.

Figure 11 and Figure 12 present the test results for compressive strength at 28 and 90 days.

The compressive strength results show similar trends to those of the flexural strength results, with the printed specimens exhibiting higher (~17%) initial strength, while molded specimens showed significantly higher (~56%) final strength. As with flexural strength, the P1% mixture exhibited the smallest difference between manufacturing methodologies, though the K1% mixture maintained greater total strength. Overall, the specimens containing cypress shavings outperformed the pine specimens by an average of 41% at the age of 28 days and by 47% at 90 days.

Testing the 3D-printed specimens parallel to the deposition layers showed, as expected, much lower compressive strength compared to perpendicular testing. At 28 days, all mixtures displayed weak interlayer cohesion, perhaps explaining the heavy fracturing under pressure that was observed during porosity testing. After 90 days of curing, there was a significant increase in strength, especially in the reference and K1% mixtures which showed 340% and 226% improvements, respectively. The P-series mixtures’ noticeably lower strength recovery suggests the poor integration of the pine wood shavings in the mortar matrix, creating weak boundary zones and compromising interlayer strength under parallel loading.

### 3.4. Stereoscopic Analysis

**Figure 13:**
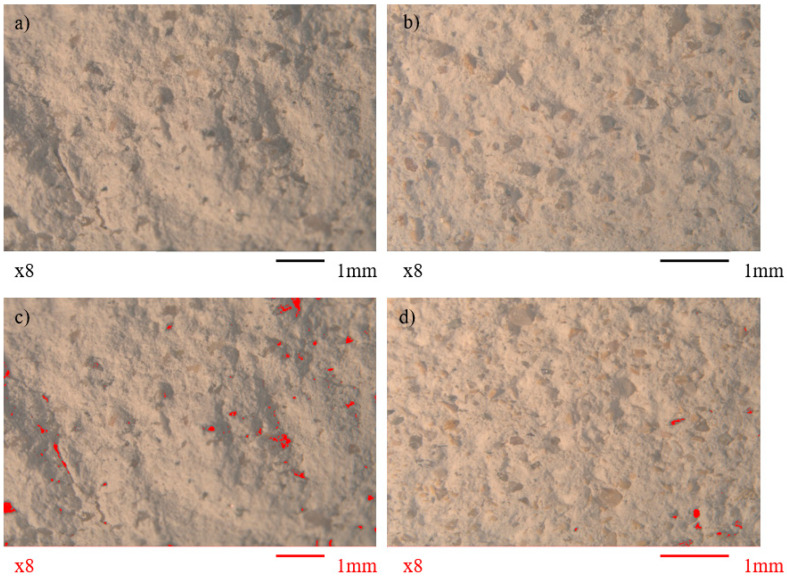
Stereoscopic images (8× magnification) of (**a**) the 3D-printed reference mixture and (**b**) the 3D-printed K3% mixture with cypress shavings (dark orange particles). In subsections (**c**,**d**), the pores present within the matrix of the mixtures are marked with red color by the Phases and Pores plugin of the DHS Image Database software (Version 2.5FEM, Dietermann and Heuser Solution GmbH, Greifenstein, Germany).

Stereoscopic observation revealed the good dispersion of aggregates and wood additives within the matrices of all printed and molded mixtures. The visible porosity ranged from ~1%, for the reference and 1% additive mixtures, to ~3%, for the 3% and 5% additive mixtures. The observed pore size started from an average of 0.34 mm for the reference mixture and progressively increased with additive inclusion to a maximum average of 0.6 and 0.65 mm for the K5% and P5% mixtures, respectively. Figure 13 illustrates the typical matrix of a 3D-printed and a molded specimen, as well as marking their visible porosity at 8× magnification.

**Figure 14:**
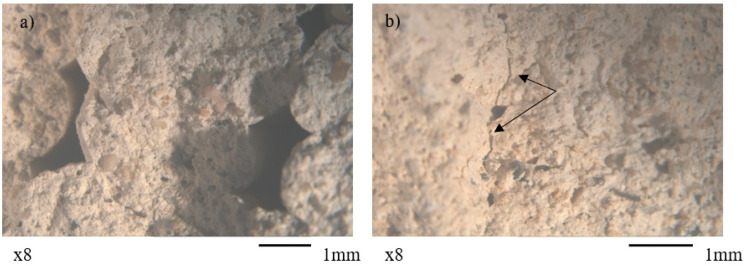
Stereoscopic images (8× magnification) of (**a**) large voids formed within the 3D-printed P5%mixture and (**b**) microfractures (arrows) within the matrix of the molded K5% mixture.

While most specimens displayed uninterrupted material distribution, the 5% additive mixtures exhibited notable structural defects for both printed and molded specimens. The stereoscopic observation of these mixtures revealed large gaps (Figure 14a) between and within the printed layers, with this effect being more pronounced at the pine mixtures where these voids measured on average 2.5 mm in length. Additionally, both printed and molded 5% specimens developed microfractures (with average length of 1 mm) throughout their matrices (Figure 14b). This void formation and microcracking suggests that the additives induced shrinkage during curing.

## 4. Conclusions

The present study introduces a methodology and proof of concept for utilizing bio-additives in traditional mortars, particularly in additive manufacturing (AM) technologies. The investigation revealed a gap in the literature regarding the incorporation of natural products in non-cement mortars for 3D printing applications. Therefore, mixtures based on lime were developed, incorporating wood shavings from pine and cypress at 1%, 3%, and 5% inclusion. Bio-additives are added to lime mortars to enhance their performance, workability, durability, and environmental impact. These materials can retain moisture longer, allowing for the better carbonation of lime and reducing shrinkage cracks. To evaluate the incorporation’s success, 3D-printed mixtures were designed and fabricated using LDM technology and compared to traditionally cast specimens.

The tests showed mixed results. On one hand, the 3D-printed specimens showed only a slight to moderate reduction in their physical and mechanical properties compared to their molded counterparts, while even improving certain properties such as early flexural and compressive strength, a known limitation of traditional mortars. However, the inclusion of wood shavings as additives caused somewhat severe degradation in the mechanical strength and cohesion of the specimens, especially at 3% and 5% concentrations. Still, the 1% mixtures, which due to the low density of the shavings still incorporated a significant volume of bio-product, displayed similar characteristics to those of the reference mixtures and can be used in applications such as the restoration of historic buildings or as indoor decorative elements. Further research should try to optimize additive–matrix bonding, interlayer cohesion, and long-term durability in order to expand the practical use of this type of mixtures. These mixtures show strong potential for further investigation and use as feedstock materials in 3D printing for the construction sector, addressing the growing need for green and sustainable materials compatible with robotic systems.

## Figures and Tables

**Table 1:** Preliminary testing of the binders and additives [32].

	Method	Hydrated Lime	Pozzolan	Cypress Wood Shavings	Pine Wood Shavings
Density (g/cm^3^)	Gas pycnometry	2.4708	2.3785	1.4398	1.4178
Particle size	LD	d (0.1): 1.216 µm d (0.5): 3.326 µm d (0.9): 16.176 µm	d (0.1): 1.498 µm d (0.5): 5.033 µm d (0.9): 33.787 µm	-	-
CaO %	XRF	87.4	1.37	-	-
MgO %	XRF	0.84	0.36	-	-
SO_3_ %	XRF	0.49	0.15	-	-
Fe_2_O_3_ %	XRF	0.08	1.34	-	-
Al_2_O_3_ %	XRF	0.03	14.60	-	-
SiO_2_ %	XRF	-	69.81	-	-
K_2_O %	XRF	-	3.02	-	-
Na_2_O %	XRF	-	2.87	-	-
TiO_2_ %	XRF	-	0.36	-	-

**Table 2:** Compositions of the experimental mortars by parts per weight.

Mortar	Mortar Abbreviation	Hydrated Lime	Pozzolan	River Sand	Water	VM	Superplasticizer	Pine Biofibers	Cypress Biofibers
Hydrated Lime–Pozzolan	REF	0.5	0.5	0.5	0.40	0.5% of binder	2% of binder	-	-
Hydrated Lime–Pozzolan–1% Pine	P1%	0.5	0.5	0.5	0.41	0.5% of binder	2% of binder	1% of binder	-
Hydrated Lime–Pozzolan–3% Pine	P3%	0.5	0.5	0.5	0.42	0.5% of binder	2% of binder	3% of binder	-
Hydrated Lime–Pozzolan–5% Pine	P5%	0.5	0.5	0.5	0.42	0.5% of binder	2% of binder	5% of binder	-
Hydrated Lime–Pozzolan–1% Cypress	K1%	0.5	0.5	0.5	0.41	0.5% of binder	2% of binder	-	1% of binder
Hydrated Lime–Pozzolan–3% Cypress	K3%	0.5	0.5	0.5	0.42	0.5% of binder	2% of binder	-	3% of binder
Hydrated Lime–Pozzolan–5% Cypress	K5%	0.5	0.5	0.5	0.42	0.5% of binder	2% of binder	-	5% of binder

## Data Availability

The original contributions presented in this study are included in the article. Further inquiries can be directed to the corresponding author.
